# A Case of *Naja atra* Bite Complicated by Gas Gangrene Leading to Amputation: Pathogen and Management Lessons

**DOI:** 10.1002/ccr3.73047

**Published:** 2026-07-05

**Authors:** Xuan Ci, Wei‐Wen Guo, Lei Yang, Yue Zhang, Zi‐Jing Liang, Jun‐Ting Huang

**Affiliations:** ^1^ Department of Blood Transfusion The First Affiliated Hospital of Guangzhou Medical University Guangzhou Guangdong Province China; ^2^ Snake Wound Department Xinhui Hospital of Traditional Chinese Medicine Jiangmen Guangdong Province China; ^3^ Department of Emergency The First Affiliated Hospital of Guangzhou Medical University Guangzhou Guangdong Province China

**Keywords:** amputation, case report, *Enterococcus faecalis*, gas gangrene, *Naja atra*

## Abstract

Gas gangrene after a *Naja atra* bite may be caused by *Enterococcus faecalis* from the snake's mouth, which is resistant to ceftriaxone. Emergency management must include early, aggressive surgical debridement and antibiotics effective against this organism (e.g., piperacillin‐tazobactam) to prevent amputation.

## Graphical abstract

Gas gangrene of the right upper limb following a Naja atra bite, with extensive skin necrosis and purulent discharge.

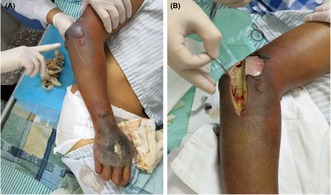

## 1 Introduction

Snakebite envenoming remains a critical global health issue, recognized by the World Health Organization as a priority neglected tropical disease. It causes significant mortality and long‐term morbidity, particularly in rural and impoverished communities where access to timely and appropriate medical care is limited [1, 2, 3]. In China, this problem is especially acute in southern regions. More than 300,000 people suffer from snakebites annually, with an estimated case fatality rate of 5% and a disability rate as high as 25%–30%, imposing a substantial burden on society and affected families [4, 5]. The Chinese cobra ( *Naja atra* ) is one of the leading causes of severe envenomation in this region.

Envenomation by *N. atra* primarily results from its potent neurotoxins and cytotoxins [6, 7]. The classic clinical syndrome involves progressive neuromuscular paralysis that often begins with ptosis and dysphagia and may advance to fatal respiratory failure. Concurrently, local cytotoxin effects lead to severe pain, swelling, blistering, and tissue necrosis at the bite site [8]. Secondary bacterial infections represent a formidable and often underestimated complication. Local tissue necrosis, occurring in over half of *N. atra* bites, creates an ideal environment for bacterial colonization [9, 10, 11]. The source of these pathogens is frequently the snake's own oral flora, which can be directly inoculated into the wound during the bite [11, 12, 13, 14]. Studies have shown that the oral cavity of *N. atra* harbors a complex microbiome, with *Enterococcus faecalis* being a predominant bacterium [11, 13, 14, 15].

Despite this knowledge, the progression from microbial inoculation to monomicrobial, life‐threatening necrotizing infection mimicking gas gangrene is poorly understood. Existing literature predominantly describes polymicrobial infections or clostridial myonecrosis, whereas the potential of monomicrobial *E. faecalis* to cause such devastating complications in snakebite wounds remains largely unreported.

Herein, we report a severe case of a patient bitten by a *Naja atra* who developed gas gangrene secondary to an *E. faecalis* infection, ultimately requiring amputation. This case underscores the virulence of the snake's oral flora and illustrates how delays in effective treatment, often associated with the use of traditional first‐aid practices, can lead to catastrophic outcomes.

## 2 Case History

A 61‐year‐old male farmer with poorly controlled hypertension, diabetes mellitus, and a history of cerebral infarction with left‐sided hemiplegia (Table 1). Presented to our emergency department 26 h after sustaining a bite from a *Naja atra* on his right hand during farming. Immediately following the bite, he experienced local pain and swelling. His condition worsened approximately 18 h post‐bite, with significant progression of swelling, exacerbated pain, and the onset of fever. He subsequently visited a local clinic, where he received cobra antivenom, dexamethasone, a gastric mucosal protective agent, an intravenous bolus of furosemide, and intravenous ceftriaxone. His symptoms failed to improve, possibly due to ceftriaxone's ineffectiveness against *E. faecalis* , and he was subsequently transferred to our hospital.

**TABLE 1:** Patient's baseline characteristics and initial presentation.

Parameter	Details
Demographics	61‐year‐old male, farmer.
Past Medical History	
Hypertension	Duration: 12 years. Control: Poor (usual BP 160/95 mmHg). Medication: Nifedipine 5 mg daily. Adherence: Irregular.
Type 2 Diabetes	Duration: 8 years. Control: Poor (last known HbA1c 9.5%). Medication: Metformin 850 mg twice daily. Adherence: Irregular.
Cerebral Infarction	3 years ago with residual left‐sided hemiplegia. Medication: Aspirin 100 mg daily.
Initial Vital Signs (on Admission)	
Temperature	38.8°C
Heart Rate	138 beats/min
Respiratory Rate	24 breaths/min
Blood Pressure	117/77 mmHg
Key Initial Laboratory Findings	
White Blood Cell Count	14.8 × 10^9^/L
Neutrophil Count	11.68 × 10^9^/L
High‐sensitivity CRP	115.2 mg/L
Creatinine	500.8 μmol/L
Blood Glucose	18.01 mmol/L
Local Wound Findings (on Admission)	Extensive necrotic area (~10 × 8 cm) on dorsum of right hand with multiple patches on forearm/elbow; subcutaneous crepitus palpable.

Upon admission, the patient was conscious but in evident distress. His vital signs were as follows: body temperature 38.8°C, pulse 138 beats/min, respiration 24 beats/min, and blood pressure 117/77 mmHg. Physical examination revealed extensive, well‐demarcated black skin necrosis (approximately 10 × 8 cm) on the dorsum of the right hand, with multiple additional necrotic patches on the forearm and elbow. The periphery of the necrotic tissue was erythematous, surrounded by severe edema and tenderness that extended to the mid‐upper arm. Subcutaneous crepitus was palpable. The blood supply to the distal extremity was initially acceptable, though the patient reported numbness. No petechiae were observed on the skin or mucous membranes.

Initial laboratory investigations revealed leukocytosis (white blood cell count 14.8 × 10^9^/L) with neutrophilia (11.68 × 10^9^/L), elevated inflammatory markers (high‐sensitivity C‐reactive protein 115.2 mg/L), coagulopathy (activated partial thromboplastin time 37.6 s; fibrinogen 8.25 g/L; D‐dimer 0.96 mg/L), and acute kidney injury (creatinine 500.8 μmol/L). Blood glucose was elevated at 18.01 mmol/L (Table 1).

**FIGURE 1:**
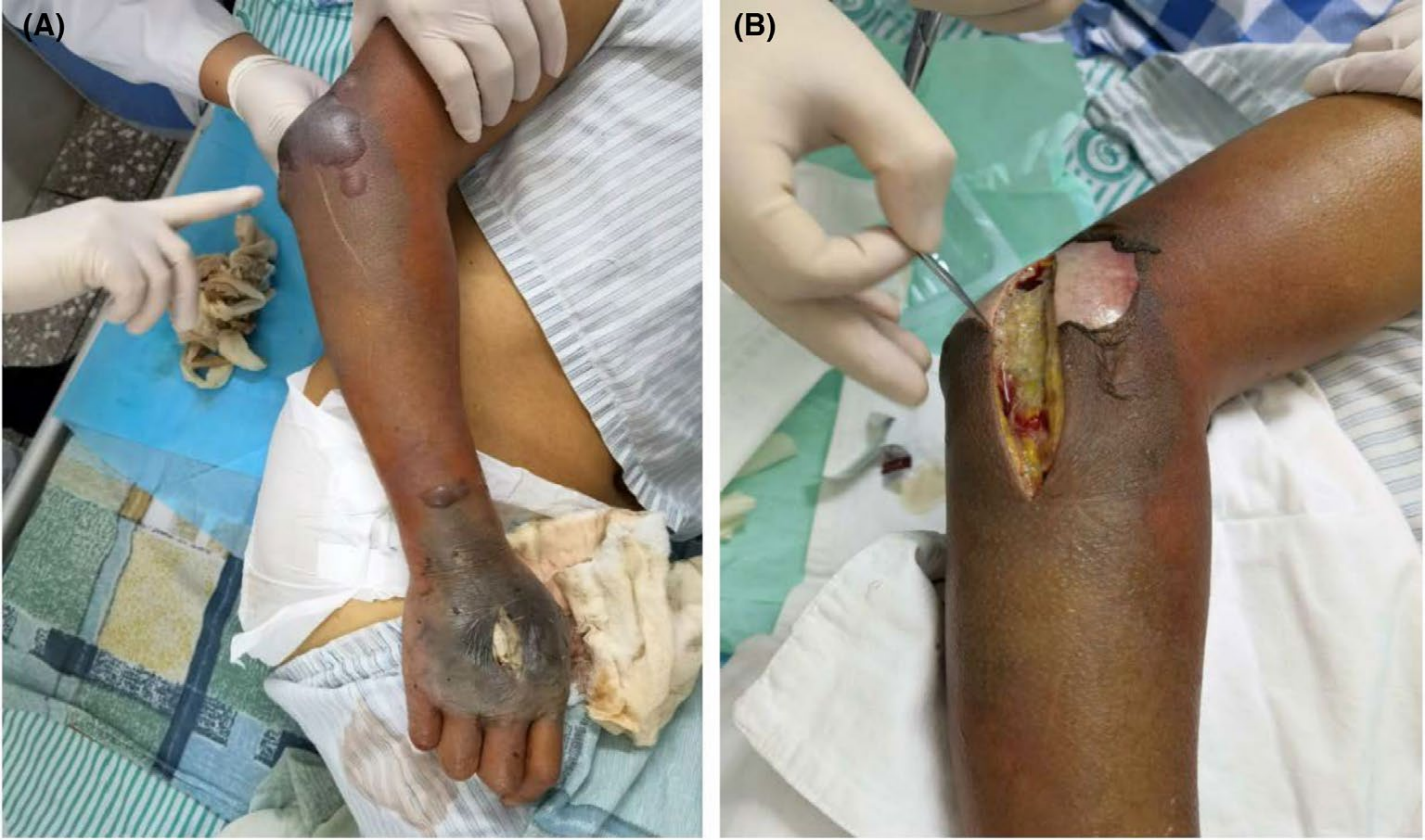
Clinical presentation of the right upper limb upon admission. (A) Extensive black skin necrosis (approximately 10 × 8 cm) on the dorsum of the hand, surrounded by severe swelling. (B) Multiple necrotic patches on the elbow and forearm; an incision has been made, draining foul‐smelling purulent discharge.

An immediate local incision and drainage were performed at the wound site, yielding approximately 50 mL of foul‐smelling, pale‐yellow purulent discharge (Figure 1). Prior to the availability of microbiological results, the patient received empirical antimicrobial coverage with cefazolin sodium targeting common skin flora, along with diuretics and tetanus immunoglobulin.

On the second hospital day, the patient's condition progressed rapidly. The necrotic areas expanded, the surrounding edema advanced proximally, and he developed severe, bursting pain. The wound produced copious serosanguinous discharge with a distinct foul odor, and subcutaneous emphysema became more prominent. A provisional diagnosis of gas gangrene was made.

Following a multidisciplinary consultation with specialists from Hand and Foot Surgery, Infectious Diseases, and Nephrology, a decision for urgent life‐saving amputation was made due to the rapidly progressive necrotizing infection. Intraoperative findings confirmed the infection had spread to the subdeltoid space. A forequarter amputation of the right upper extremity was performed. The wound was irrigated copiously with a sequential solution of povidone‐iodine, hydrogen peroxide, and normal saline. Postoperatively, given the rapidly worsening condition and suspected gas‐forming infection, the antibiotic regimen was escalated to broad‐spectrum coverage (piperacillin‐tazobactam and meropenem) active against enterococci and anaerobes, combined with vacuum‐sealing drainage and continuous gentamicin sulfate irrigation.

**FIGURE 2:**
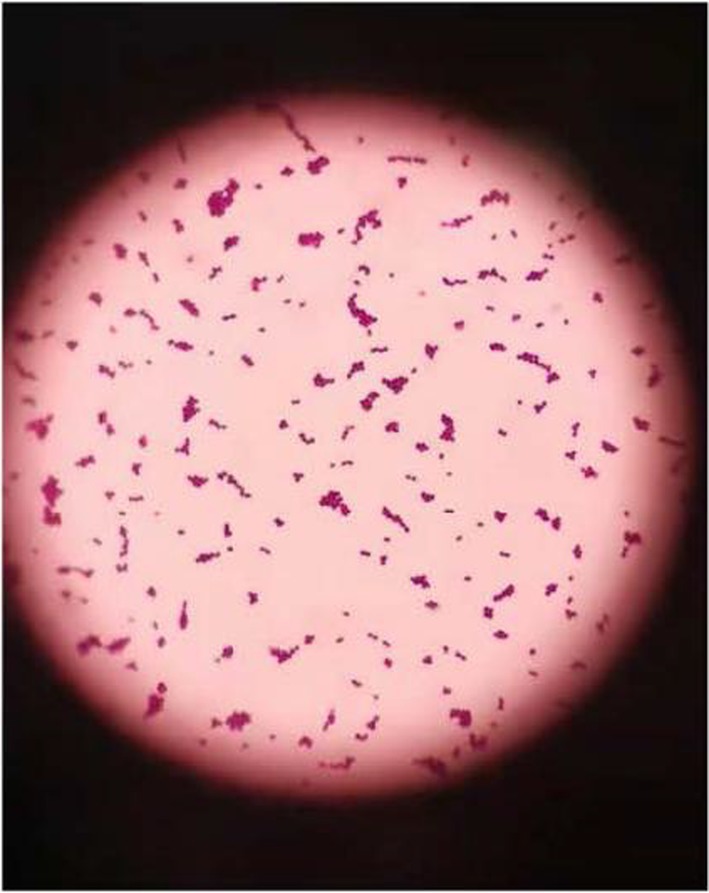
Microbiological analysis of wound exudate. Gram staining (100× oil immersion objective) revealed Gram‐positive cocci arranged in pairs and short chains, consistent with the morphology of *Enterococcus faecalis* , which was subsequently confirmed by culture.

**FIGURE 3:**
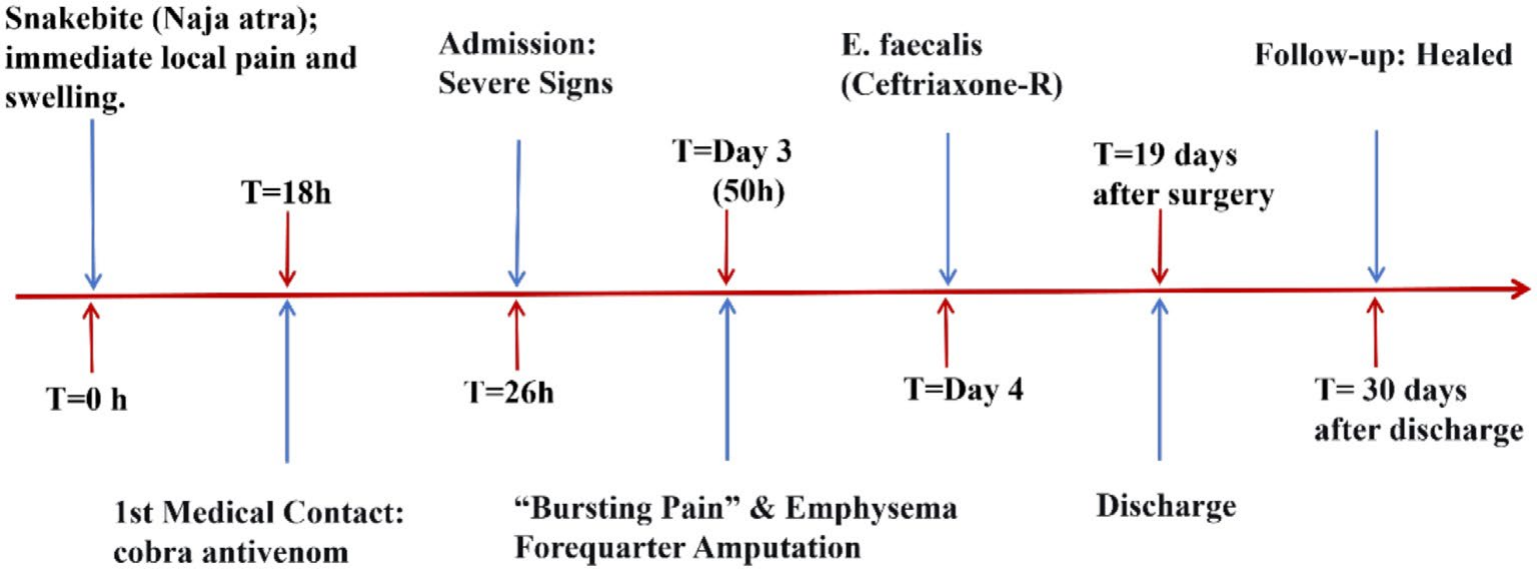
Timeline of the clinical course from snakebite to surgery.

Bacterial culture results from the necrotic wound and incision site, obtained 3 days post‐admission, confirmed monomicrobial growth of *E. faecalis* in all specimens (Figure 2 and Table 2). The patient's condition gradually stabilized with targeted antibiotic therapy and wound care. He was successfully discharged on postoperative day 19. At a follow‐up visit 1 month after discharge (approximately 49 days postoperatively), the amputation stump was well‐healed with no signs of infection recurrence, and he had begun prosthetic rehabilitation (Figure 3). The patient provided written informed consent for the publication of the clinical details and images associated with his case.

**TABLE 2:** In vitro antibiotic susceptibility profile of the *Enterococcus faecalis* isolate from the wound swab.

Antibiotic	Susceptibility (S/R/I)	MIC (μg/mL)	Method
Levofloxacin	S	0.25	MIC
Linezolid	S	2	MIC
Tigecycline	S	≤ 0.12	MIC
Tetracycline	S	≤ 1	MIC
Ampicillin	S	≤ 2	MIC
Ciprofloxacin	S	≤ 0.5	MIC
Erythromycin	S	0.5	MIC
Nitrofurantoin	S	≤ 16	MIC
Vancomycin	S	1	MIC
Penicillin‐G	S	4	MIC
Gentamicin High Level Synergy	S	SYN‐S	MIC
Streptomycin High Level synergy	S	SYN‐S	MIC

*Note:* Bacterial count: ++ (Moderate growth).

Abbreviations: I, Intermediate; MIC, Minimum inhibitory concentration; R, Resistant; S, Susceptible; SYN‐S, Synergy‐Susceptible (indicating suitability for combination therapy with a cell‐wall active agent).

## 3 Differential Diagnosis

Following a snakebite, the patient presented with a necrotic wound, crepitus, and systemic toxic symptoms, leading to a primary consideration of necrotizing soft tissue infection. The differential diagnoses include (1) Clostridial myonecrosis (classic gas gangrene). Typically caused by *Clostridium perfringens* , characterized by rapid progression, severe pain, and marked crepitus. While the progression rate in this case was consistent, the absence of typical ‘dishwater‐like’ discharge during surgery and the final culture showing monomicrobial *E. faecalis* growth did not support this diagnosis [16]. (2) Polymicrobial necrotizing fasciitis: Commonly seen in contaminated wounds, involving a mixture of aerobic and anaerobic bacteria. Although snakebite wounds are inherently contaminated, all specimens in this case yielded monomicrobial growth of *E. faecalis* , essentially ruling out the typical polymicrobial pattern. (3) Progressive local tissue necrosis due to cobra venom cytotoxins: The venom can cause local tissue necrosis but is not typically accompanied by copious purulent discharge, foul odor, or significant subcutaneous emphysema. The prominent infectious signs in this case (high fever, pus, emphysema) indicated that a severe secondary bacterial infection was the dominant factor [6, 7].

## 4 Discussion

This case report describes a severe instance of a *Naja atra* bite that was complicated by a secondary *E. faecalis* infection leading to gas gangrene, ultimately necessitating amputation. It highlights two critical factors influencing the outcome: the snake's oral flora and inappropriate initial management.

The isolation of *E. faecalis* in this case of severe wound infection is consistent with its recognized role as a predominant bacterium in the oral cavity of *Naja atra* [17, 18, 19]. As a hardy, facultative anaerobic Gram‐positive coccus, *E. faecalis* exhibits intrinsic resistance to many commonly used antibiotics, such as cephalosporins, and thrives in diverse environments, including necrotic wounds with compromised blood supply [20]. While gas gangrene is primarily caused by anaerobic Clostridium species [16]. *E. faecalis* is frequently implicated in polymicrobial necrotizing infections following cobra bites, where it contributes to the complex infectious landscape [21, 22, 23]. Its ability to form biofilms and synergize with other bacteria enhances its pathogenicity in mixed infections. While classic gas gangrene is typically caused by Clostridium species, this case demonstrates that in the necrotic tissue resulting from a snakebite, resident oral bacteria such as *E. faecalis* can also trigger rapidly progressive necrotizing infections accompanied by subcutaneous emphysema. This underscores the necessity of obtaining bacterial cultures early to identify the pathogen in snakebite management, rather than relying solely on presumptive diagnosis.

A review of the treatment course revealed several key missteps. Firstly, the initial application of herbal remedies likely created a localized, hypoxic environment that promoted bacterial proliferation. Secondly, the ceftriaxone administered at the local clinic was inherently ineffective against *E. faecalis* due to its natural resistance to most cephalosporins [24]. Rendering the initial antibiotic therapy inadequate. Finally, and most critically, the delay of over 24 h from the bite to definitive surgical intervention allowed the infection to spread extensively into the subdeltoid space, leaving amputation as the only life‐saving measure to control the infection. This reaffirms that for established necrotizing infections, prompt and radical surgical debridement is the cornerstone of management and cannot be substituted by pharmacological treatment alone [25, 26, 27].

This case uniquely demonstrates that monomicrobial *E. faecalis* can cause fulminant gas gangrene following a *Naja atra* bite, a presentation scarcely reported in contrast to the typical polymicrobial or clostridial infections [21, 22, 23]. Based on the lessons from this case, the management of *Naja atra* bites with severe local manifestations mandates an integrated approach: immediate empiric antibiotic therapy with agents like piperacillin‐tazobactam that are effective against the snake's oral flora, which includes a broad spectrum of Gram‐negative and Gram‐positive pathogens [15]; a low threshold for early and aggressive surgical debridement upon signs of systemic toxicity or rapid progression [28]; and comprehensive supportive care including meticulous wound management and multidisciplinary collaboration [25].

## 5 Conclusion

This single‐center case report has inherent limitations. The late presentation precluded early anaerobic cultures, potentially missing initial microbial dynamics. Initial management was influenced by prehospital factors, which may have altered the disease course. Nevertheless, this case holds valuable cautionary significance for clinical practice. The snake's oral *E. faecalis* can act as an accomplice in causing severe secondary infections. Delays in care due to factors like poverty and traditional practices provide a breeding ground for such infections. Therefore, maintaining a high index of suspicion, initiating empiric broad‐spectrum antibiotic coverage targeting the oral flora, and setting a low threshold for early and aggressive surgical intervention are crucial to preventing such tragic outcomes.

## Author Contributions

**Xuan Ci:** data curation, writing – original draft. **Wei‐Wen Guo:** data curation, writing – original draft. **Lei Yang:** data curation, investigation. **Yue Zhang:** resources. **Zi‐Jing Liang:** project administration. **Jun‐Ting Huang:** writing – review and editing.

## Funding

This work was supported by the Basic and Applied Basic Research Foundation of Guangdong Province (ID: 2022A1515012095) and 2021 Guangzhou Science and Technology Program City and University (Hospital) joint funded Grant (ID: 202102010282).

## Consent

Written informed consent was obtained from the patient for the publication of this case report, including all clinical details and accompanying images.

## Conflicts of Interest

The authors declare no conflicts of interest.

## Data Availability

The data that support the findings of this study are available on request from the corresponding author. The data are not publicly available due to privacy or ethical restrictions.
